# Breast cancer radiotherapy in Sub-Saharan Africa: a comparative study of acute toxicity between conventional and hypofractionated treatment regimens

**DOI:** 10.3332/ecancer.2024.1810

**Published:** 2024-12-05

**Authors:** Joseph Daniels, Tony Obeng-Mensah, Kofi Adesi Kyei

**Affiliations:** 1 National Centre for Radiotherapy, Oncology and Nuclear Medicine, Korle Bu Teaching Hospital, PO Box KB 369, Korle Bu, Accra, Ghana; 2 Department of Radiography, University of Ghana, Legon, PO Box KB 143, Korle Bu, Accra, Ghana; a https://orcid.org/0000-0002-1466-150X; b https://orcid.org/0000-0003-3485-5368

**Keywords:** breast cancer, hypofractionation, acute radiation-induced toxicity, radiotherapy, conventional fractionation

## Abstract

Hypofractionated radiotherapy for breast cancer has been increasingly adopted globally due to its comparable efficacy and reduced treatment burden. The study compared the incidence and severity of four main acute radiation-induced toxicities between breast cancer patients treated with conventional versus hypofractionated radiotherapy. Stratified purposive sampling was used to recruit participants into two groups: group #1 received conventional radiotherapy (50 Gy in 25 fractions over 5 weeks), whereas group #2 received hypofractionated radiotherapy (40.05 Gy in 15 fractions over 3 weeks). A closed-ended questionnaire administered by the researcher was used for quantitative data collection. The Common Terminology Criteria for Adverse Events tool (version 5) was used for grading acute toxicities. Data were analyzed using the Statistical Package for Social Sciences (version 23). The study involved 53 patients with a mean age of 47.9 years (± 12.4) ranging from 26 to 75 years. The patients had breast cancer ranging from stage IIA (13.2%) to IIIC (9.4%). A considerable majority (62.3%) were treated with conventional fractionation whereas 37.7% were treated with a hypofractionated regimen. Dermatitis was the most prevalent side effect among patients in both groups #1 (67%) and #2 (70%). There were no statistically significant differences in the incidence of dermatitis, pharyngitis, chest wall/ breast pain and fatigue between the two groups. However, the mean incidence of overall acute toxicity was significantly lower in group #2 (2.15 ± 1.14) compared with group #1 (2.42 ± 1.48), with a p-value of 0.001. Comparatively, the conventional 50 Gy dose regimen was associated with more acute radiation-induced toxicity.

## Introduction

Breast cancer is the most frequently diagnosed cancer and the leading cause of cancer-related mortality among females worldwide, presenting a significant public health challenge, especially in resource-limited settings. According to recent estimates, 2.3 million new cases of breast cancer were diagnosed globally in 2022, with 670,000 associated fatalities [1]. In Sub-Saharan Africa, the incidence of breast cancer has been rising, with many patients presenting with advanced stages of the disease [2]. Contributing factors to this trend include erroneous symptom interpretation, belief in the potency of alternative medicine, lack of trust in orthodox medicine and limited healthcare access [3]. Breast cancer management involves a combination of surgery, chemotherapy, radiotherapy, hormonal and targeted therapy, depending on the stage and immunohistochemical characteristics of the tumour. The use of radiotherapy reduces local recurrence and improves survival rates [4]. However, the choice of radiotherapy regimen can significantly impact both short- and long-term toxicity profiles, particularly in resource-limited settings.

Radiation treatments are typically offered in divided doses known as fractions, to reduce radiation-induced side effects experienced by normal tissues [5]. Fractionation of the radiation dose produces, in most cases, better tumour control for a given level of normal tissue toxicity than a single large dose [8]. The conventional fractionation regimen for treating breast cancer is 50 Gy delivered in 25 daily fractions of 2 Gy each (from Monday to Friday each week), over a period of 5 weeks [6]. Although effective, it can be logistically challenging for patients in sub-Saharan Africa due to the need for prolonged daily travel to treatment centres and the associated costs [7]. Shorter, hypofractionated regimens delivering a total dose of 42.56 Gy in 16 fractions or 40.05 Gy in 15 fractions, have been prescribed for cases of early breast cancer [5]. The use of ultra-hypofractionated regimens, such as delivering 26 Gy in 5 fractions either daily within 1 week or once weekly over 5 weeks, has been increasingly adopted due to its advantages in reducing treatment burden for patients and optimizing radiotherapy equipment use, especially in settings with high patient volumes. These regimens are also appealing for their convenience and potential cost savings, particularly in resource-constrained environments. Studies like the UK FAST-Forward trial have demonstrated that this approach is as effective and safe as traditional regimens, promoting its adoption globally [8]. Professional guidelines from international organizations such as the European Society for Radiotherapy and Oncology – Advisory Committee in Radiation Oncology Practice have supported the use of ultra-hypofractionated regimens to improve patient access to timely care [9].

Studies have shown that hypofractionation provides similar efficacy to conventional fractionation, with comparable local control and survival rates [5, 6]. Hypofractionated radiotherapy has been increasingly adopted in high-resource settings due to its convenience and comparable efficacy [5]. Recent studies have also demonstrated the feasibility and effectiveness of hypofractionated radiotherapy in resource-limited settings. Studies in Nigeria have demonstrated that hypofractionated regimens lead to comparable clinical outcomes with a reduction in acute skin toxicity and treatment duration [10, 11].

Radiotherapy to the breast or chest wall after (breast) surgery is associated with several side effects, which can be classified as either acute or late effects. Acute side effects including skin reactions (dermatitis), fatigue and breast pain, can significantly affect the quality of life of patients with breast cancer undergoing radiation treatment [12]. Late (long-term) effects of breast cancer radiotherapy include telangiectasia, secondary malignancies, pulmonitis and subcutaneous fibrosis. A study by Hussein and Al-Rawq [13] indicated that the commonest acute side effects among breast cancer patients were radiation-induced dermatitis, fatigue, chest wall/breast pain, pharyngitis, nausea and dysphagia.

Despite promising results, the adoption of hypofractionated radiotherapy in sub-Saharan Africa has been slow, partly due to a lack of robust comparative data on its acute toxicity profile in these settings.

There is a paucity of published literature addressing the incidence of acute side effects regarding fractionation regimens used for breast cancer radiotherapy in sub-Saharan. The study compared the incidence and severity of acute radiation-induced toxicities among breast cancer patients treated with conventional versus hypofractionated radiotherapy regimens at a major radiotherapy centre in Ghana.

## Methods

### Study design and setting

This research was a descriptive quantitative cross-sectional study conducted at a major radiotherapy and cancer treatment centre in west Africa. The centre plays a key role in providing radiation therapy services to patients across Ghana and neighboring countries. The centre delivers external beam radiotherapy using a cobalt-60 teletherapy machine and a 6 MV linear accelerator. Other services offered include palliative care, patient education and support, survivorship care, cancer screening chemotherapy, hormonal and targeted therapy as well as immunotherapy.

### Participants

The study population comprised adult female patients diagnosed with breast cancer who underwent adjuvant external beam radiotherapy at the study site over a 5-month period. Only patients with a confirmed histological diagnosis of invasive breast cancer were included in the study, excluding those with ductal carcinoma *in situ* or breast sarcoma. Patients with metastatic or inoperable breast cancer receiving palliative radiotherapy were also not included in the study. The study included only eligible patients who provided written informed consent. A stratified purposive sampling technique was used to recruit consenting eligible participants into the study. The study population was initially stratified into two groups. Group #1 comprised eligible patients receiving conventional radiotherapy (50 Gy in 25 fractions of 2 Gy per fraction delivered over 5 weeks) whereas group #2 comprised participants who were treated with hypofractionated radiotherapy (40.05 Gy in 15 fractions of 2.67 Gy per fraction delivered over 3 weeks). Subsequently, eligible participants were purposively selected from each group based on the inclusion and exclusion criteria.

### Study size

The annual load of new breast cancer patients managed with radiotherapy at the study site is about 500 patients including those with inoperable disease or de novo metastasis to distant sites. The proportion of patients in this population treated with curative intent is about two thirds. Based on the monthly throughput of patients with breast cancer treated with adjuvant external beam radiotherapy at the centre, the appropriate sample size for the study was determined to be 70 patients, per Yamane’s formula [14]. Over the study period, only a limited number of patients met the strict inclusion criteria and were available for participation. Multiple episodes of treatment machine breakdown occurred during the study period, hampering the recruitment of patients into the study. Ethical considerations, such as obtaining informed consent, further reduced the potential pool of participants. In total, 53 patients were recruited in this study, 33 participants in group #1 and 20 in group #2.

### Variables

#### Breast cancer staging

Breast cancer stage grouping was based on the 8th edition of the American Joint Committee on Cancer staging system [15]. Stage 0, is also known as carcinoma *in situ* and represents non-invasive cancers like ductal carcinoma *in situ*. Stage IA incorporates tumour size ≤2 cm, without lymph node involvement or distant metastasis whereas stage IB comprises tumours of similar size with small cancer cell clusters (0.2–2 mm) found in the lymph nodes, though there is no distant metastasis. Stage IIA refers to tumours measuring between 2 and 5 cm without lymph node spread, or smaller than 2 cm but with small lymph node involvement. In Stage IIB, the tumour is 2–5 cm with spread to nearby lymph nodes or larger than 5 cm without lymph node involvement. In Stage IIIA, the tumour can be of any size with cancer spread to 4–9 axillary lymph nodes. Stage IIIB is characterized by tumour spread to the chest wall or skin, with possible lymph node involvement whereas stage IIIC involves cancer spread to 10 or more axillary lymph nodes or other nearby nodes, such as the infraclavicular nodes. Stage IV breast cancer indicates distant metastasis, meaning cancer has spread to organs such as the bones, liver, lungs or brain, regardless of the primary tumour size or lymph node involvement.

#### Mean incidence of overall acute toxicity

The ‘mean incidence’ represented the average number of distinct acute toxicity events experienced by each patient, across the different types of toxicities (e.g., dermatitis, fatigue, pharyngitis and breast/chest wall pain). For each patient, the presence of any of these toxicities was scored (1 if present, 0 if absent), and the total score for all toxicities was summed to generate an overall acute toxicity score per patient (maximum score of 4, i.e., if the patient experienced all four distinct acute toxicity events). These individual scores were then averaged across the entire cohort in both the conventional and hypofractionated radiotherapy groups to calculate the ‘mean incidence of overall toxicity’.

### Data collection

Data were collected using a structured two-sectioned questionnaire (Appendix 1) that was administered by the researcher. The first section gathered demographic information, while the second section focused on the occurrence and severity of four main acute radiation-induced side effects: dermatitis, fatigue, pharyngitis and breast/chest wall pain. Patients were followed up for 12 weeks (3 months) after completion of radiotherapy to assess the peak of acute side effects. The severity of these side effects was graded using the Common Terminology Criteria for Adverse Events (CTCAE) grading scale (version 5.0) [16]. Patients’ hospital-based medical records were also accessed for clinical as well as tumour- and treatment-related information including cancer stage, fractionation regimen and type of surgery. The research instrument was validated by a consultant radiation oncologist and a senior radiation therapist. A pilot study with eight participants was conducted to assess the reliability and clarity of the questionnaire. While the questionnaires were primarily in English, they were translated into relevant local dialects as needed.

### Bias

The study utilized a structured two-section questionnaire designed to ensure consistency in the data collected from all participants. This approach reduced the variability that could arise from open-ended questions and subjective interpretations, thereby minimizing response bias. The questionnaire was administered by the researcher, which allowed for immediate clarification of any questions or uncertainties that participants might have had. This direct interaction helped ensure that respondents understood the questions as intended, further reducing the risk of misunderstanding and misreporting. Conducting a pilot study allowed the researcher to identify and address any potential biases in the questionnaire itself. Feedback from this pilot testing informed refinements to the questionnaire, ensuring it accurately captured the necessary data without leading or biased questions.

### Statistical methods

Data were analyzed using the Statistical Package for Social Sciences (SPSS) version 23. Descriptive statistics were used to summarize baseline patient characteristics, including demographics, tumour characteristics and treatment details. Continuous variables were reported as means and standard deviations whereas categorical variables were expressed as frequencies and percentages. The incidence of acute toxicity was compared across treatment arms using the chi-square test with statistical significance set at a *p*-value <0.05.

### Ethical considerations

This study was approved by the institutional ethical and protocol review committee (SBAHS-RD./10571661/SA/2022). The study was conducted according to established ethical guidelines and in accordance with the ethical standards laid down in an appropriate version of the 2000 Declaration of Helsinki as well as the Declaration of Istanbul 2008. Privacy and confidentiality of patient information were maintained throughout the study. All participants provided written informed consent prior to their inclusion in the study.

## Results

### Baseline characteristics

**Figure 1.**
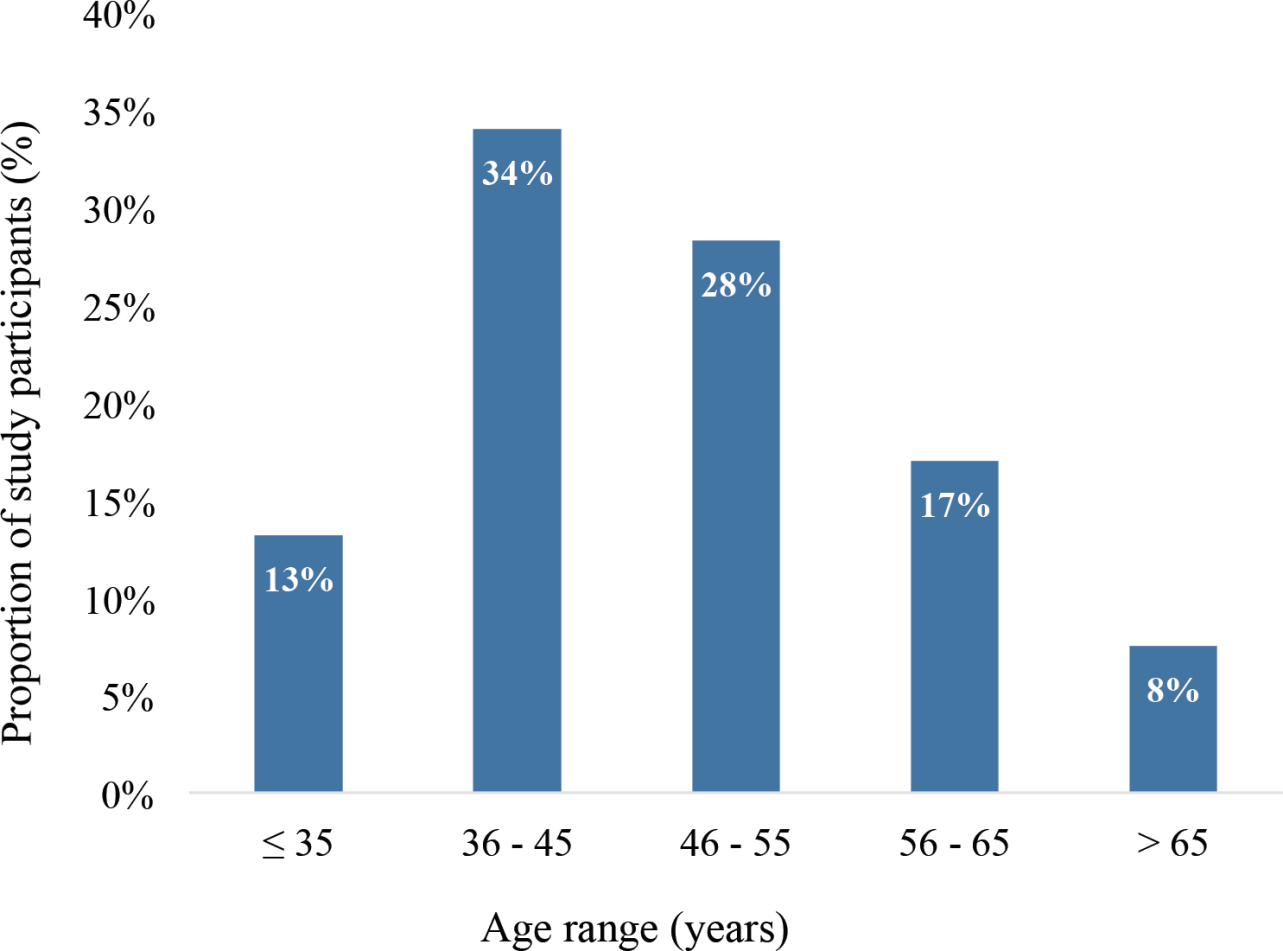
Age distribution of study participants (*N* = 53).

A total of 53 adult female breast cancer patients participated in this study. Their age distribution is depicted in Figure 1. In all, 34% of the patients were between 36 and 45 years whereas 28% were between 46 and 55 years. Also, 13% were 35 years or younger whereas 8% were 65 years or older. The group mean age was 47.9 years (± 12.4) ranging from 26 to 75 years.

The laterality of the affected breast, stage, treatment regimen (dose fractionation), type of surgery and treatment fields used are summarized in Table 1. There was an equal distribution of left- and right-sided breast cancers, 26 (49.1%) and 27 (50.9%), respectively. Overall, the participants had breast cancer ranging from stage IIA (*n* = 7, 13.2%) to stage IIIC (*n* = 5, 9.4%). Also, 17 participants (32.1%) had stage IIB disease whereas 14 (26.4%) had stage IIIB breast cancer. In total, 37 patients (69.8%) underwent a modified radical mastectomy whereas 16 (30.2%) underwent breast-conserving surgery in the form of a wide local excision with ipsilateral levels I and II axillary lymph node dissection. The patients who underwent breast-conserving surgery were treated with two tangential fields to the whole breast with (*n* = 12, 22.6%) or without (*n* = 4, 7.5%) regional nodal (supraclavicular) irradiation. On the other hand, the patients who underwent mastectomy were treated with two tangential fields to the chestwall, also with (*n* = 30, 56.6%) or without (*n* = 7, 13.3%) irradiation of the supraclavicular region as shown in Table 1. Additionally, 33 participants (62.3%) were treated with conventional fractionation whereas 20 (37.7%) were treated with the hypofractionated regimen.

### Incidence of acute radiation-induced toxicity

**Figure 2.**
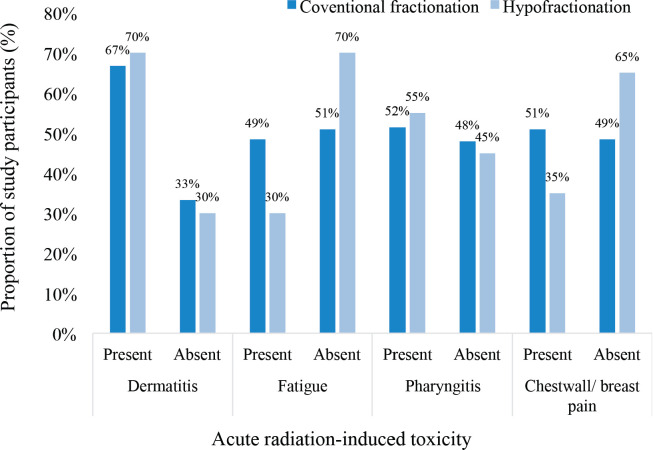
Incidence of acute radiation-induced toxicities.

Among participants treated with conventional fractionation, the most frequently occurring acute radiation-induced toxicity was dermatitis (67%), followed by pharyngitis (52%), breast/chest wall pain (52%) and fatigue (49%). On the other hand, among those treated with the hypofractionated regimen, the commonest acute toxicity was dermatitis (70%), followed by pharyngitis (55%), chestwall/ breast pain (35%) and fatigue (30%) as illustrated in Figure 2.

### Severity of acute radiation-induced toxicities

**Figure 3.**
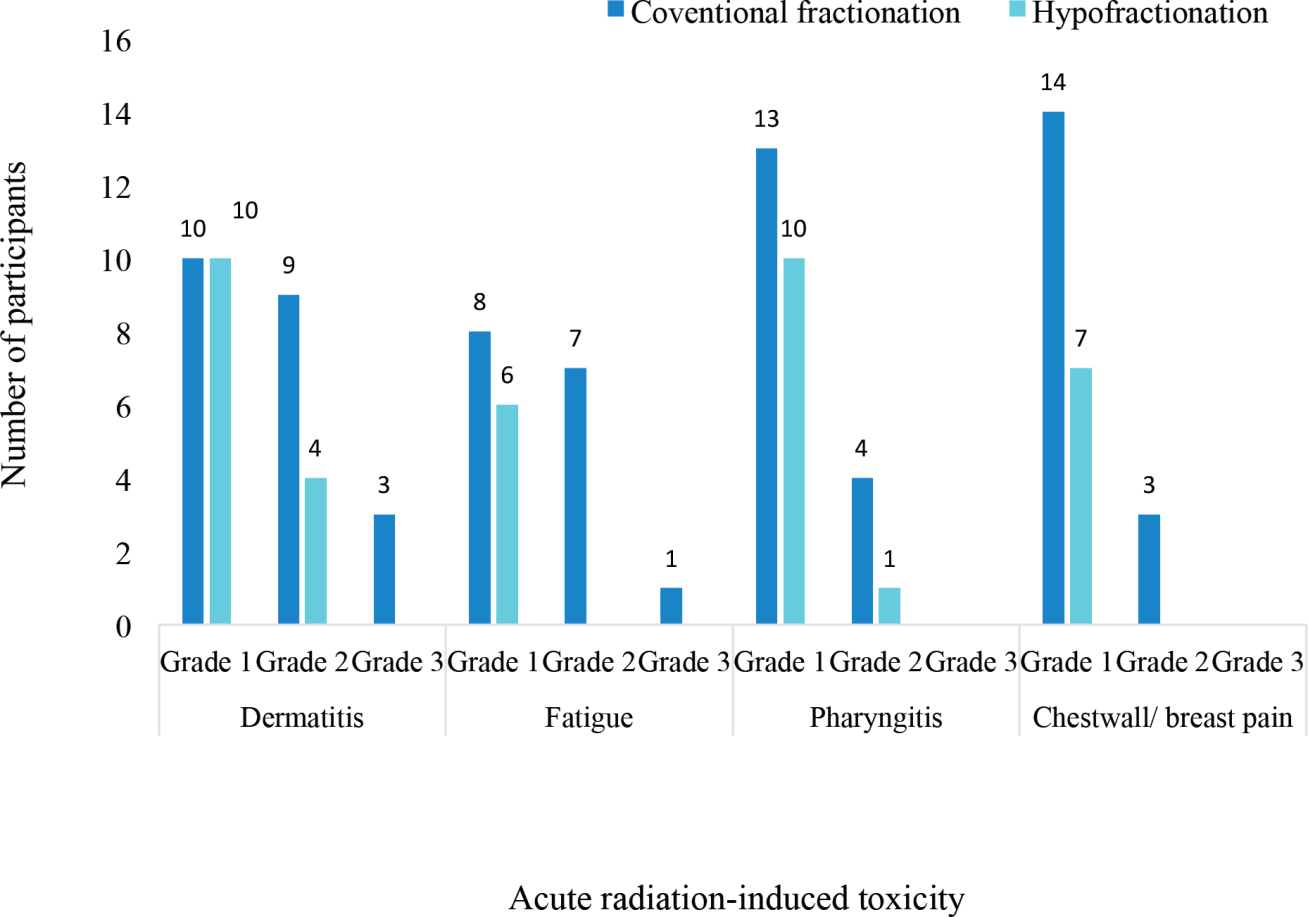
Severity of acute radiation-induced toxicities.

Different grades of radiation-induced toxicities were recorded among the patients treated with both conventional and hypofractionated regimens. 10 patients each had grade 1 dermatitis among the participants treated with conventional fractionation and those treated with hypofractionation. The pattern of incidence of the different CTCAE grades of the four selected acute radiation-induced toxicities are illustrated in Figure 3. There were no grade 4 or 5 toxicities recorded among the patients. None of the patients had either grade 3 pharyngitis or chestwall/ breast pain.

### Chi square analysis of acute radiation-induced toxicities

Table 2 presents the statistical analysis of acute toxicity outcomes and the mean incidence of side effects between conventional and hypofractionated radiotherapy regimens. Chi-square tests indicated no statistically significant differences in the incidence of specific acute toxicities, including dermatitis (*p* = 0.801), fatigue (*p* = 0.186), pharyngitis (*p* = 0.805) and breast/chest wall pain (*p* = 0.421), between the two radiotherapy regimens. However, the mean incidence of overall acute toxicity was significantly lower in the hypofractionated group (2.15 ± 1.14) compared to the conventional fractionation group (2.42 ± 1.48), with a *p*-value of 0.001. This suggests that while individual acute toxicities may not differ significantly, the overall burden of acute toxicity is less with hypofractionated radiotherapy.

## Discussion

The study investigated the incidence and severity of acute radiation-induced toxicities among breast cancer patients treated with either conventional fractionation or hypofractionated radiotherapy regimens. In all, 53 female patients participated in this study with a mean age of 47.9 years (± 12.4) ranging from 26 to 75 years. There were equal proportions of left- (49.1%) and right-sided (50.9%) breast cancers with no case of bilateral breast cancer in either treatment group. Overall, the participants had breast cancer ranging from stage IIA (13.2%) to stage IIIC (9.4%). Also, 69.8% underwent mastectomy whereas 30.2% underwent breast-conserving surgery. In all, 62.3% were treated with conventional fractionation whereas 37.7% were treated with a hypofractionated regimen. The most common acute radiation-induced toxicity in group #1 was dermatitis (67%), followed by pharyngitis (52%), breast or chest wall pain (52%) and fatigue (49%). In group #2, dermatitis was also the most prevalent acute toxicity, affecting 70%, followed by pharyngitis (55%), chest wall or breast pain (35%) and fatigue (30%). There were no statistically significant differences in the incidence of these acute toxicities. However, the mean incidence of overall acute toxicity was significantly lower in the hypofractionated group (2.15 ± 1.14) compared to the conventional fractionation group (2.42 ± 1.48), *p*-value = 0.001.

The study population had a mean age of 47.9 years, which is slightly younger than the average age of breast cancer patients in high-income countries, where the median age is typically over 60 years [17]. In a similar study on the qualitative analysis of acute skin toxicity among breast cancer radiotherapy patients, Schnur *et al* [18] reported an age range of 38–84 years and a mean population age of 60 ± 12.4 years. There were more breast cancer patients in the middle age brackets (36–45 and 46–55 age groups) in this study, which is consistent with the findings of Kyei *et al* [19] who reported that in Ghana, breast cancer frequently affects female patients aged more than 40 years. Breast cancer patients in high-income countries tend to be older than their counterparts in resource-limited settings partly because of differences in life expectancy [20]. The younger age of the participants may have influenced the incidence of certain toxicities, such as skin reactions, which can be more severe in younger patients due to higher skin elasticity and cell turnover rates (Lee [21] *et al*., 2020).

There were similar proportions of both right and left-sided breast cancers. However, according to Petrovich [22] breast cancer is more likely to be diagnosed in the left breast. The predominance of mastectomy (69.8%) in this cohort reflects treatment patterns in sub-Saharan Africa, where breast-conserving surgery is less common due to delayed presentation and more advanced disease at diagnosis [23]. Only 13.2% of the patients presented with stage IIA disease. The majority (*n* = 29, 54.7%) presented with advanced disease in the IIIA, IIIB and IIIC stage groups, consistent with the general pattern of late presentation among breast cancer patients in the subregion [3]. Factors such as lack of trust and confidence in orthodox medicine and limited access to healthcare may account for this trend [24].

According to the literature, radiotherapy is comparatively well tolerable in patients with breast cancer. Using current technology, the therapeutic ratio has been considerably enhanced, while the potential for side effects has considerably decreased [25]. No patient developed severe side effects that required treatment interruption; however, each patient experienced at least one radiotherapy-induced toxicity (CTCAE grade 1 to 3). Dermatitis was the most prevalent acute radiation-induced toxicity in both the conventional (67%) and hypofractionated (70%) groups, which is consistent with findings from similar studies conducted in both high- and low-income settings [26]. In a large multicentre cohort study, Jagsi *et al* [27] reported a 60% incidence grade 2 dermatitis in patients receiving breast radiotherapy whereas another study by Hussein and Al-Rawaq [13] found a 58% incidence of grade 2 and 3 in breast cancer patients treated using intensity-modulated radiotherapy. Dermatitis is a well-documented side effect of breast cancer radiotherapy, particularly in patients receiving treatment to larger breast volumes or those with fairer skin [28]. However, in this study, there were no significant differences in the incidence of specific acute toxicities between the two treatment groups, which aligns with global trends showing similar toxicity profiles for both regimens [29]. It remains imperative for healthcare providers to explore the use of cost-effective topical treatments, preventive skincare regimens and other supportive care measures specifically tailored for breast cancer patients undergoing radiotherapy in low-resource settings. Such strategies could significantly improve patient comfort, reduce the severity of acute skin toxicity and enhance overall treatment adherence and outcomes. Additionally, incorporating these interventions into routine care protocols in resource-limited environments could alleviate the burden on healthcare systems while enhancing the quality of life for patients.

Pharyngitis and chest wall pain were also common in both groups, with pharyngitis slightly more prevalent in the hypofractionated group (55% versus 52%). This finding supports the hypothesis that while hypofractionation may reduce treatment time, it does not necessarily diminish the frequency of certain acute toxicities [30]. The lower incidence of chest wall pain in the hypofractionated group (35%) compared to the conventional group (52%) may suggest a potential benefit of hypofractionation in terms of reducing patient discomfort, though this finding was not statistically significant. Fatigue was the least frequent early side effect (41.5%). In the study of Jagsi *et al* [27] the incidence of acute fatigue side effects was in the range of 60%–96%.

Although the incidence of specific toxicities did not differ significantly between the two groups, the overall severity of acute toxicities, as measured by the mean incidence, was significantly lower in the hypofractionated group (2.15 ± 1.14) compared to the conventional group (2.42 ± 1.48). This finding is clinically significant and suggests that hypofractionation may offer a safer toxicity profile in terms of overall burden on the patient. This supports the notion that hypofractionated radiotherapy is not only effective but also offers a better toxicity profile, making it a preferable option for some patients. Overall, these findings suggest that hypofractionation could be a more patient-friendly approach with fewer acute toxicities without compromising efficacy.

Studies in high-income settings have consistently shown that hypofractionation is associated with comparable or even lower toxicity rates compared to conventional fractionation. For instance, a study in Canada by Whelan [31] found that acute skin toxicity was significantly reduced in patients receiving hypofractionated treatment. These findings are mirrored in resource-limited settings, such as India and South Africa, where hypofractionation has been shown to maintain efficacy while potentially reducing acute side effects [32, 33]. In sub-Saharan Africa, where access to radiotherapy is often limited by equipment availability and patient load, the adoption of hypofractionated regimens could offer a practical solution to these challenges. By shortening the duration of treatment, hypofractionation can increase the number of patients treated, reduce the strain on radiotherapy resources and improve patient outcomes [33]. This study adds valuable data to the limited body of literature on the safety and feasibility of hypofractionation in this region.

## Limitations

The CTCAE grading of the severity of the radiation-induced toxicities experienced by the patients was retrieved from their medical records as documented by their treating physicians. This information could not be independently verified by the researchers. Even though the study was conducted at the largest radiotherapy centre in Ghana, generalizing the findings to other regions within sub-Saharan Africa should be done with caution. The sample size (53 patients) was relatively small, which may have reduced the power of the study to detect subtle differences in the incidence of specific toxicities between the two treatment groups. Another limitation of the study involves the potential bias introduced by the validation of the research instrument. While the questionnaire was reviewed by a consultant radiation oncologist and a senior radiation therapist to ensure clinical relevance, the reliance on only two experts may have introduced biases based on their personal experiences and clinical practices. Also, the study focused only on acute toxicity and hence, long-term side effects were not assessed.

## Conclusion

The most common acute toxicity associated with radiotherapy were acute dermatitis from radiation, pharyngitis, pain in the irradiated region and fatigue. The study demonstrates that the hypofractionated radiotherapy regimen of 40.05 Gy in 15 fractions delivered over 3 weeks, is associated with a significantly lower incidence of acute toxicities compared to the conventional regimen of 50 Gy in 25 fractions delivered over 5 weeks in breast cancer patients in Ghana. These findings are consistent with global trends and suggest that hypofractionation could be a valuable tool for improving breast cancer care in sub-Saharan Africa. These findings support the use of hypofractionation as a more tolerable and efficient treatment option for breast cancer, particularly in settings where minimizing treatment burden and resource utilization is critical.

## Recommendations

The adoption of hypofractionated breast cancer radiotherapy, specifically the regimen of delivering 40.05 Gy in 15 fractions over 3 weeks, should be prioritized as it is associated with a significantly lower incidence of acute toxicities compared to conventional regimens. This approach aligns with global trends and highlights its potential to enhance patient comfort and treatment efficiency. Healthcare facilities in sub-Saharan Africa should also consider integrating hypofractionation into their standard treatment protocols, which could lead to better patient outcomes and more efficient use of radiotherapy resources. Collaborative research and multicentre trials are recommended to further validate these findings and optimize treatment strategies across diverse populations. Patient education should emphasize the benefits of hypofractionated radiotherapy to improve adherence and satisfaction, ensuring that the advantages of hypofractionation are fully realized. It is also important to explore the development and implementation of effective mitigation strategies to manage acute radiation-induced toxicities, particularly dermatitis, which was the most common side effect observed in this study. Further research should investigate the long-term toxicities of hypofractionation and explore the adoption of ultra-hypofractionated regimens, such as delivering 26 Gy in 5 fractions either once weekly over 5 weeks or daily, within 1 week.

## Conflicts of interest

The authors declare no competing interest.

## Funding

This study did not receive any specific funding support from funding agencies in the public, commercial or not-for-profit sectors.

## Data availability

The data used to support the findings of this study are available from the corresponding author upon reasonable request.

## Figures and Tables

**Table 1.** Clinical and treatment characteristics of the study participants (*N* = 53).

Characteristics	Variables	Number (*N*)	Percentage (%)
**Laterality of the affected breast**	Left	26	49.1
	Right	27	50.9
*Cancer stage group	IIA	7	13.2
	IIB	17	32.1
	IIIA	10	18.9
	IIIB	14	26.4
	IIIC	5	9.4
**Type of surgery**	Mastectomy	37	69.8
	Breast conservation surgery	16	30.2
**Fractionation regimen**	Conventional# (50Gy/25#/2Gy)	33	62.3
	Hypo# (40.05Gy/ 15#/ 2.67Gy)	20	37.7
**Treatment fields**	Chest wall only	7	13.3
	Chest wall + supraclav	30	56.6
	Whole breast only	4	7.5
	Whole breast + supraclav	12	22.6

Conventional#: conventional fractionation, hypo#: hypofractionation, #: fractions, supraclav: supraclavicular region. *The stage grouping was based on the 8^th^ edition of the American Joint Committee on Cancer staging system [15]

**Table 2.** Chi square analysis of acute radiation-induced toxicities.

Acute toxicity	*χ* ^2^		*p*-value
Dermatitis	0.64		0.801
Fatigue	1.75		0.186
Pharyngitis	0.61		0.805
Breast/Chest wall pain	0.65		0.421
**Fractionation regimen**	*Mean incidence	**±**	***p*-value**
Conventional fractionation	2.42	1.48	0.001
Hypofractionation	2.15	1.14	

* This represents the mean incidence of overall acute toxicity
